# Low-Energy-Loss Polymer Solar Cells with 14.52% Efficiency Enabled by Wide-Band-Gap Copolymers

**DOI:** 10.1016/j.isci.2018.12.027

**Published:** 2019-01-06

**Authors:** Kui Feng, Jian Yuan, Zhaozhao Bi, Wei Ma, Xiaopeng Xu, Guangjun Zhang, Qiang Peng

**Affiliations:** 1 Key Laboratory of Green Chemistry and Technology of Ministry of Education, College of Chemistry, State Key Laboratory of Polymer Materials Engineering, Sichuan University, Chengdu 610064, PRC; 2 State Key Laboratory for Mechanical Behavior of Materials, Xi'an Jiaotong University, Xi'an 710049, PRC

**Keywords:** Chemical Synthesis, Energy Materials, Devices

## Abstract

Two wide band-gap copolymers poly[4,8-bis(5-(2-butylhexylthio)thiophen-2-yl)benzo[1,2-b:4,5-b′]dithiophene-2,6-diyl-*alt*-TZNT] (PBDTS-TZNT) and poly[4,8-bis(4-fluoro-5-(2-butylhexylthio)thiophen-2-yl)benzo[1,2-b:4,5-b′]dithiophene-2,6-diyl-*alt*-TZNT] (PBDTSF-TZNT) based on naphtho[1,2-c:5,6-c]bis(2-octyl-[1,2,3]triazole) (TZNT) and benzo[1,2-b:4,5-b']dithiophene (BDT) with different conjugated side chains have been developed for efficient nonfullerene polymer solar cells (NF-PSCs). The rigid planar backbone of BDT and TZNT units imparted high crystallinity and good molecular stacking property to these copolymers. Using 3,9-bis(2-methylene-(3-(1,1-dicyanomethylene)-indanone)-5,5,11,11-tetrakis(4-hexylphenyl)-dithieno[2,3-d:2′,3′-d′]-s-indaceno[1,2-b:5,6-b′]-dithiophene (ITIC) as the acceptor, PBDTSF-TZNT devices showed a high *V*_oc_ of 0.98 V with an E_loss_ of 0.61 eV. On selecting 3,9-bis(2-methylene-(5,6-difluoro-(3-(1,1-dicyanomethylene)-indanone)-5,5,11,11-tetrakis(4-hexylphenyl)-dithieno[2,3-d:2′,3′-d′]-s-indaceno[1,2-b:5,6-b’]-dithiophene (IT-4F) instead of ITIC, the devices maintained the high *V*_oc_ of 0.93 V with an even lower E_loss_ of 0.59 eV. The combination of the above-mentioned low E_loss_, broadened absorption, better matched energy level, improved crystallinity, and fine-tuned morphology promoted the power conversion efficiency (PCE) of PBDTSF-TZNT:IT-4F devices from 12.16% to 13.25%. Homo-tandem devices based on PBDTSF-TZNT:IT-4F subcells further enhanced the light-harvesting ability and boosted the PCE of 14.52%, which is the best value for homo-tandem NF-PSCs at present.

## Highlights

• High-performance TZNT-based wide-band-gap copolymers were designed and synthesized
• The rigid planar backbone induced high crystallinity and good molecular packing
• Sulfuration and fluorination fine-tuned HOMO aiming for high V_oc_ and low E_loss_
• Single-junction and homo-tandem devices exhibited high PCEs of 13.25% and 14.52%

## Graphical Abstract

**Figure:**
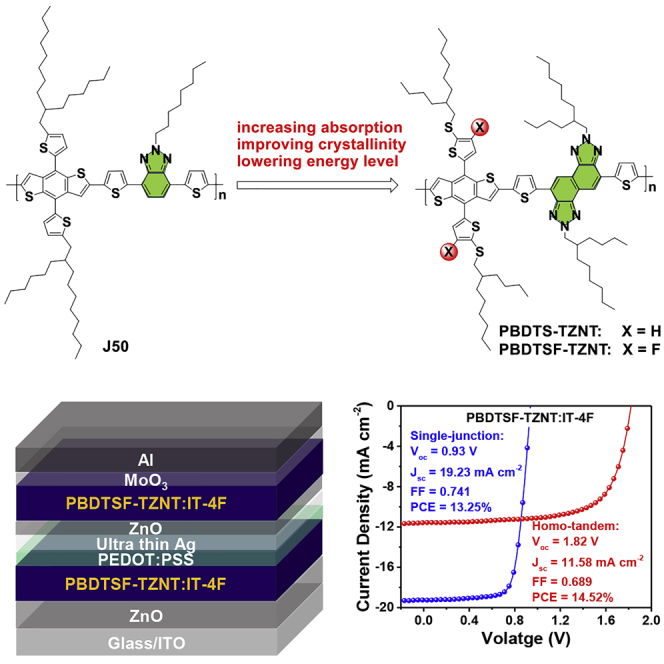

## Introduction

Polymer solar cells (PSCs), which contain a nanophase-separated bicontinuous network of a *p*-type conjugated polymer donor and an *n*-type semiconductor acceptor, have received considerable attention owing to their unique prospects for achieving low cost, light weight, and mechanical flexibility in solar energy applications (Cheng et al., 2009, Li et al., 2012, Zhang et al., 2018b). During the past few years, nonfullerene acceptors (NFAs) have aroused intense interest because of the synthetic flexibility and great potential to overcome the intrinsic drawbacks of the fullerene counterparts (Lin and Zhan, 2016, Yan et al., 2018, Zhang et al., 2018b). Particularly, planar acceptor-donor-acceptor (A-D-A)-type NFAs presented low band gaps (LBGs) with good absorptions, readily tunable energy levels, and superior photovoltaic performance (Cui et al., 2017, Li et al., 2016, Lin et al., 2015a, Lin et al., 2015b, Lin et al., 2016, Lin and Zhan, 2016, Xu et al., 2017, Yan et al., 2018, Zhang et al., 2018b). State-of-the-art power conversion efficiencies (PCEs) exceeding 13% for single-junction nonfullerene PSCs (NF-PSCs) (Li et al., 2018a, Xiao et al., 2017, Zhang et al., 2018c, Zhang et al., 2018d, Zheng et al., 2018) and 17% for tandem NF-PSCs (Meng et al., 2018) have been realized, showing a bright future for practical applications.

To make full use of the present excellent LBG NFAs, developing high-performance wide-band-gap (WBG) donor copolymers has become a hot research topic (Cai et al., 2017). WBG copolymers have strong absorption in the short wavelength region, which can match well with LBG NFAs to realize a complementary absorption and then an improved photocurrent in NF-PSCs (Xu et al., 2018a, Xu et al., 2018b). Besides the issue of complementary absorption, low-lying highest occupied molecular orbital (HOMO) level and appropriate molecular aggregation of the WBG donor are equally important for achieving high PCE because the HOMO level and molecular packing can directly influence the open-circuit voltage (*V*_oc_) and charge carrier mobility (Cai et al., 2017, Li et al., 2018a, Xu et al., 2018b). Among the efficient material systems of WBG copolymers, benzotriazole (BTA)-based donor-acceptor copolymers have received wide research interests. Such types of copolymers provide an advantage of incorporating soluble alkyl chains onto the BTA skeleton rather than on the thiophene bridges of other WBG copolymers, which can also reduce the steric repulsion between the adjacent segments for more planar π-conjugations (Min et al., 2011, Price et al., 2011). Therefore, BTA-based copolymers exhibited excellent solubility, strong absorption, and high charge carrier mobility. Extensive studies combining side chain engineering and fluorination strategies have been made (Bin et al., 2016a, Bin et al., 2016b, Min et al., 2012, Price et al., 2011, Xue et al., 2017), and have increased the PCE to over 12% (Lin et al., 2018, Liu et al., 2018, Luo et al., 2018, Zhao et al., 2017a, Zhao et al., 2018). With respect to the above-mentioned molecular design, extending the π-conjugation of the building blocks has been proved to be positive for more ordered molecular packing, improved intramolecular charge transfer transition, enhanced absorption, and thus elevated photovoltaic performance (Dong et al., 2013, Osaka and Takimiya, 2017, Wang et al., 2011, Yu et al., 2017). Based on this, naphtho[1,2-c:5,6-c]bis(2-octyl-[1,2,3]triazole) (TZNT) was developed to construct high-performance donor copolymers, which fused two BTA units with an angular shape (Dong et al., 2013). Apart from the advantages discussed above, the enhanced electron deficit of TZNT could also lower the HOMO level of the resulting copolymers, contributing to high *V*_oc_s and PCEs in fullerene PSCs (Dong et al., 2013, Lan et al., 2015). Apart from the above two reports, most recently, a high PCE of over 10% for a new TZNT-based copolymer of PDTF-TZNT has been first realized in NF-PSCs by our group (Tang et al., 2018b). However, the relatively high HOMO level (−5.24 eV) caused by the strong electron-donating donor block of thiophene led to low *V*_oc_ (0.8 V) and large energy loss (E_loss_ = 0.8 eV, defined as E_loss_ = E_g_^opt^ – q*V*_oc_, where E_g_^opt^ is the optical band gap and q is the elementary charge). To further increase the *V*_oc_ and reduce the E_loss_, the electron-donating strength of the incorporated donor blocks should be weakened.

In terms of light harvesting, it is still a great challenge for achieving panchromatic absorption by just using a single active layer due to the limited absorption ability. A successful and universal strategy to elevate the device performance is to use tandem configurations. Tandem solar cells connect multiple photoactive layers by highly transparent interconnecting layers (Shim et al., 2012, Vasilopoulou et al., 2015), offering more efficient light-harvesting ability than single-junction solar cells due to enhanced light trapping without sacrificing charge transport property, which can induce high *V*_oc_ and PCE (Li et al., 2013, Zuo et al., 2014).

**Figure 1:**
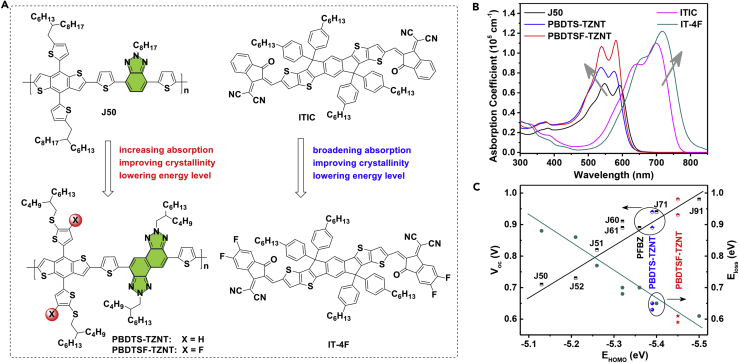
Design of Wide-Band-Gap Copolymer Donors with Low-Lying HOMO Level (A) Molecular structures of the copolymers and NFAs. (B) Absorption profiles of the copolymers and NFAs. (C) Summarized *V*_oc_ and E_loss_ versus E_HOMO_.

**Scheme 1:**
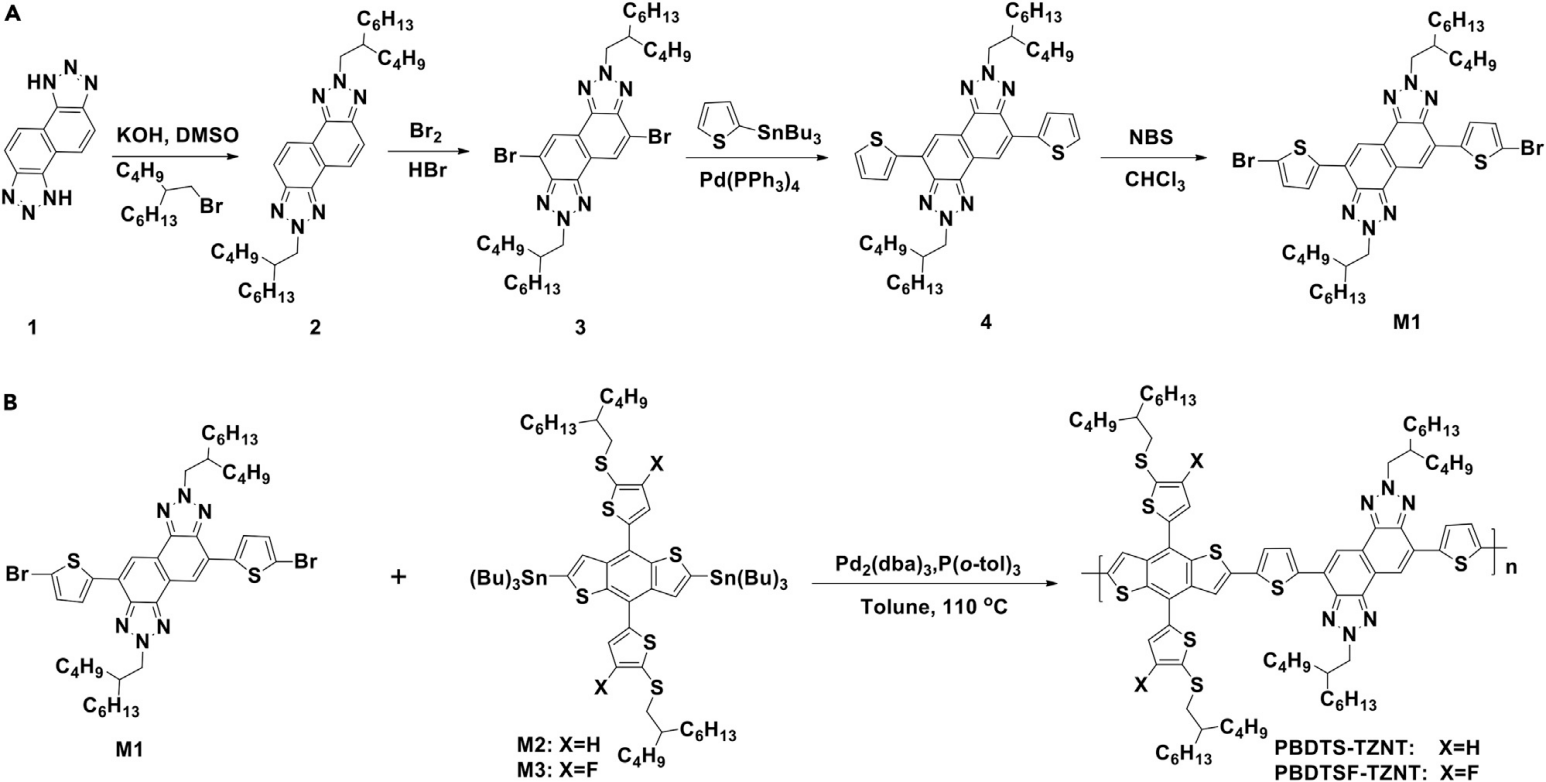
Synthetic Routes of the Monomers and Copolymers (A) Synthetic route of monomer M1. (B) Synthetic routes of PBDTS-TZNT and PBDTSF-TZNT. Also see Figure S1.

Herein, we report the design and synthesis of two novel WBG copolymers, poly[4,8-bis(5-(2-butylhexylthio)thiophen-2-yl)benzo[1,2-b:4,5-b′]dithiophene-2,6-diyl-*alt*-TZNT] (PBDTS-TZNT) and poly[4,8-bis(4-fluoro-5-(2-butylhexylthio)thiophen-2-yl)benzo[1,2-b:4,5-b′]dithiophene-2,6-diyl-*alt*-TZNT] (PBDTSF-TZNT), based on TZNT and benzo[1,2-b:4,5-b']dithiophene (BDT) blocks for realizing highly efficient NF-PSCs (Figure 1 and Scheme 1). BDT unit, a promising weak electron-donating donor block, possesses a rigid and large coplanar structure for inducing a strong π-π stacking (Ye et al., 2014). Energy level, absorption spectrum, crystallinity, charge carrier mobility, and blend morphology can be feasibly tailored by manipulating the two-dimensional (2D) conjugated side chains of BDT (Bin et al., 2016a, Bin et al., 2016b, Fan et al., 2017, Fan et al., 2018, Fei et al., 2018, Li et al., 2018b, Tang et al., 2018a, Xu et al., 2018b, Xue et al., 2017, Ye et al., 2014, Zhang et al., 2018a, Zhang et al., 2018c, Zhao et al., 2017b). Aiming to lower the E_loss_ and realize high performance of TZNT-based WBG polymers here, BDT derivatives with alkylthiothienyl group and fluorinated alkylthiothienyl side chains were selected as the donor blocks. As expected, these two copolymers exhibited a band gap approaching 2.0 eV, which could enable complementary absorption to those excellent LBG acceptors. The HOMO level lowered from −5.39 eV for PBDTS-TZNT to −5.45 eV for PBDTS-TZNT by introducing sulfur atoms or both sulfur and fluorine atoms. Such low HOMO level of PBDTSF-TZNT contributed a very large *V*_oc_ of 0.98 V if using 3,9-bis(2-methylene-(3-(1,1-dicyanomethylene)-indanone)-5,5,11,11-tetrakis(4-hexylphenyl)-dithieno[2,3-d:2′,3′-d′]-s-indaceno[1,2-b:5,6-b′]-dithiophene (ITIC) as the acceptor, leading to low E_loss_ of 0.61 V and high PCE of 12.16%. Moreover, the large *V*_oc_ of 0.93 V could be maintained even using 3,9-bis(2-methylene-(5,6-difluoro-(3-(1,1-dicyanomethylene)-indanone)-5,5,11,11-tetrakis(4-hexylphenyl)-dithieno[2,3-d:2′,3′-d′]-s-indaceno[1,2-b:5,6-b′]-dithiophene (IT-4F) acceptor with a much lower lowest unoccupied molecular orbital (LUMO) level (−3.99 eV) (Zhang et al., 2018c), which further lowered the E_loss_ to 0.59 eV and improved the PCE up to 13.25%. To further enhance the light-harvesting ability and elevate the device performance, homo-tandem devices containing two PBDTSF-TZNT:IT-4F-based subcells were fabricated. The resulting tandem devices displayed a very high PCE of 14.52% with a *V*_oc_ of 1.82 V, a short current density (*J*_sc_) of 11.58 mA cm^−2^, and a fill factor (FF) of 68.9%, which is the best record for homo-tandem NF-PSCs reported at present.

## Results and Discussion

The synthetic routes of the intermediates and target copolymers are shown in Scheme 1. Their detailed synthesis procedures are provided in the Supplemental Information. The organic tin monomers (M2 and M3) and naphtha[1,2-c:5,6-c]bis(1H-[1,2,3]triazole) (compound 1) were synthesized according to previous procedures (Dong et al., 2013, Zhang et al., 2018a). Compound 3 was synthesized by alkylation of compound 1 and bromination of compound 2. Compound M1 was synthesized by Stille coupling of compound 3 with tributyl(4-(2-butyloctyl)thiophen-2-yl)stannane then followed by bromination. The target copolymers of PBDTS-TZNT and PBDTSF-TZNT were synthesized by Stille coupling polymerization using Pd_2_(dba)_3_/P(*o*-tolyl)_3_ as catalyst in good yields. The intermediates and the final products were characterized by nuclear magnetic resonance spectroscopy and elemental analysis. Both copolymers have good solubility in common organic solvents, such as chloroform (CF), toluene, chlorobenzene (CB), and *o*-dichlorobenzene. The number average molecular weights (*M*_n_s) were 43.1 and 44.5 kDa, and the polydispersity indices were 2.48 and 2.51 for PBDTS-TZNT and PBDTSF-TZNT, respectively, determined by gel permeation chromatography. The decomposition temperatures (*T*_d_s, 5% weight loss) were 343°C and 356°C for PBDTS-TZNT and PBDTSF-TZNT, respectively, showing their good thermal stabilities (Figure S1A). Differential scanning calorimetry experiments displayed no obvious endothermal and exothermal peaks from room temperature to 300°C (Figure S1B), which might be due to the rigid backbones of the copolymers that limited the chain motion (Osaka et al., 2012).

**Figure 2:**
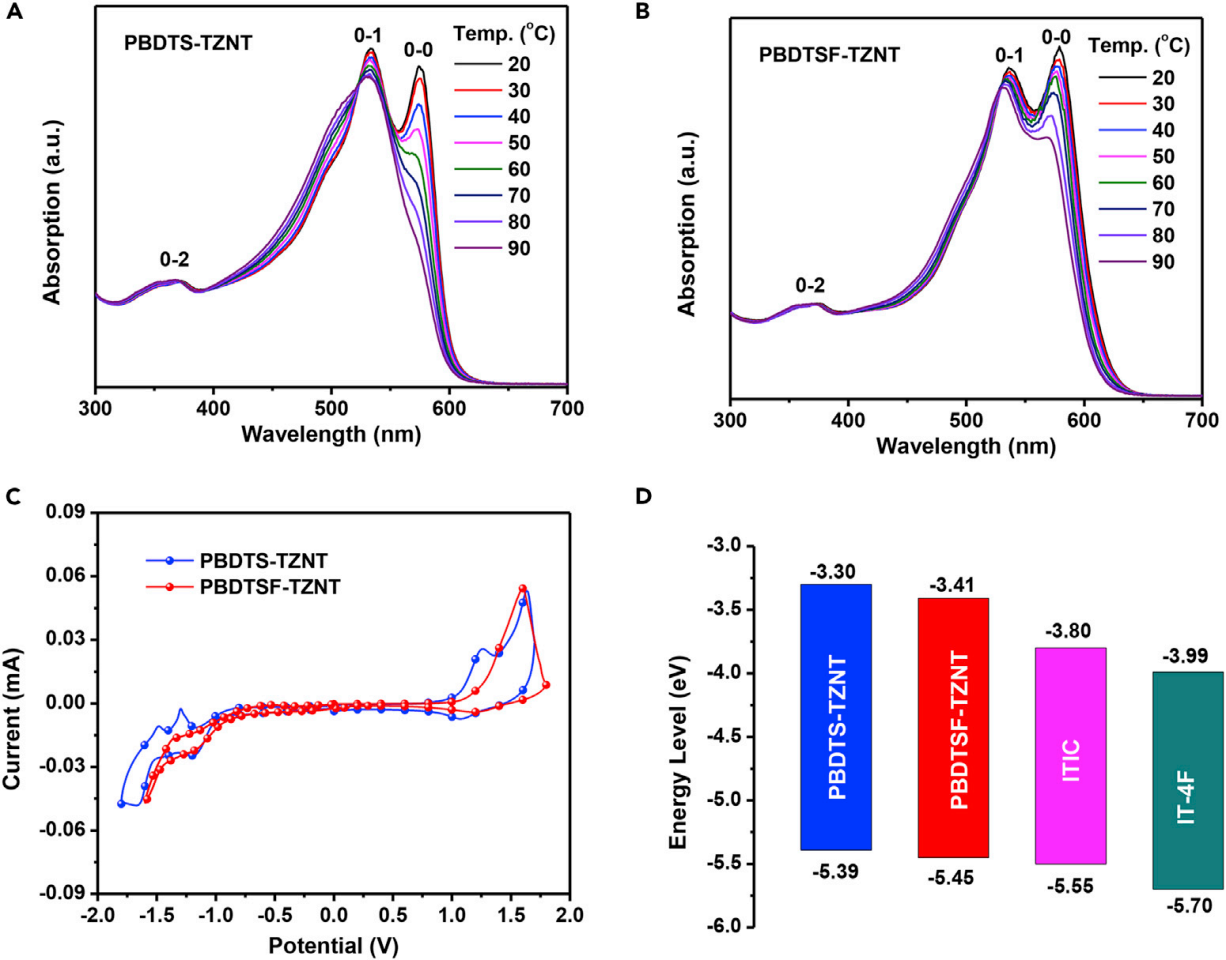
Absorption and Energy Level Comparison of Copolymer Donors and Acceptors (A) The temperature-dependent absorption of PBDTS-TZNT in CB solution. (B) The temperature-dependent absorption of PBDTSF-TZNT in CB solution (C) Cyclic voltammogram curves of PBDTS-TZNT and PBDTSF-TZNT. (D) Energy level diagram of the donor and acceptor materials. Also see Figures S2 and S3.

Figures 2A and 2B showed the ultraviolet-visible absorption spectra of these two copolymers in CB solution at different temperatures. Both copolymers displayed similar absorption profiles with an obvious peak shoulder at room temperature due to their identical backbones. The 0-0, 0-1, and 0-2 peaks were assigned to the inter-, intramolecular, and π-π* transitions, respectively (Wan et al., 2017). Compared with PBDTS-TZNT, the slightly red-shifted 0-0 (579 versus 574 nm) and 0-1 (536 versus 533 nm) peaks demonstrated stronger molecular aggregation. Also, this was further examined by increasing the solution temperature. The peak 0-0 peak of PBDTS-TZNT decreased sharply with increasing the temperature, and almost disappeared at high temperature. However, such peak shoulder could be still observed in PBDTSF-TZNT solution even at 90°C. The stronger aggregation of PBDTSF-TZNT would help to enhance the absorption and crystallinity. In film states, the 0-0 and 0-1 peaks were 576 and 537 nm for PBDTS-TZNT and 582 and 540 nm for PBDTSF-TZNT, respectively (Figure 1B). Compared with their solution counterparts, the red-shifted absorption indicated that the molecular packing was enhanced in film state. The maximum absorption coefficient was 8.54 × 10^4^ cm^−1^ (537 nm) for PBDTS-TZNT and 1.12 × 10^5^ cm^−1^ (540 nm) for PBDTSF-TZNT, respectively (Figure 1B). In contrast, J50, which incorporated the BTA block, exhibited a much lower maximum absorption coefficient of 6.79 × 10^4^ cm^−1^ (Figure 1B). J91 with both fluorination on the BTA skeleton and the conjugated side chains of BDT showed a much higher absorption coefficient (0.98 × 10^5^ cm^−1^) than J50, but still lower than our newly prepared copolymer of PBDTSF-TZNT (Xue et al., 2017). The stronger absorption of PBDTSF-TZNT would be beneficial for harvesting more photons in devices. The optical band gaps (E_g_^opt^s) of PBDTS-TZNT and PBDTSF-TZNT were 1.99 and 1.97 eV, respectively, which could form good complementary absorption with typical LBG NFAs, such as ITIC and IT-4F (Figures 1B and S2). The optical characteristics of the copolymers are summarized in Table 1.

**Table 1:** Optical and Electrochemical Properties of the Copolymers

Copolymer	Solution	Film	E_g_^opt^ (eV)	HOMO (eV)	LUMO (eV)	E_g_^CV^ (eV)
	λ_peak_ (nm)	λ_peak_ (nm)				
PBDTS-TZNT	533,575	537.576	1.99	−5.39	−3.30	2.09
PBDTSF-TZNT	536,579	540,582	1.97	−5.45	−3.41	2.04

Cyclic voltammogram was employed to estimate the energy levels of the copolymers (Figure 2C). The HOMO/LUMO levels of PBDTS-TZNT and PBDTSF-TZNT were −5.39/-3.30 and −5.45/-3.41 eV, respectively, which matched well with ITIC and IT-4F acceptors (Figure 2D) (Zhang et al., 2018c). The lower-lying HOMO level of PBDTSF-TZNT could be expected to obtain higher *V*_oc_ in devices (Figure 1C). Moreover, the very low HOMO offset between PBDTSF-TZNT and ITIC (0.07 eV) or IT-4F (0.22 eV) could also be beneficial for reducing the E_loss_ to further increase *V*_oc_. Density functional theory calculations were performed at the B3LYP/6-31G(d) level to investigate the electronic structures of these two copolymers. As shown in Figure S3, the fluorinated side chains on PBDTSF-TZNT showed a smaller dihedral angle of 53.7° than that of PBDTSF-TZNT (54.1°) in the ground state, which agreed well with the absorption differences as discussed above. The densities of their HOMOs were more populated on the BDT segments, whereas the LUMOs were mainly distributed over the TZNT skeletons. The HOMO/LUMO levels of PBDTS-TZNT and PBDTSF-TZNT were calculated to be −4.76/-2.51 and −4.85/-2.61 eV, respectively, which agreed well with the electrochemical results, indicating that fluorination could efficiently lower the energy levels but had less effect on the band gaps.

**Figure 3:**
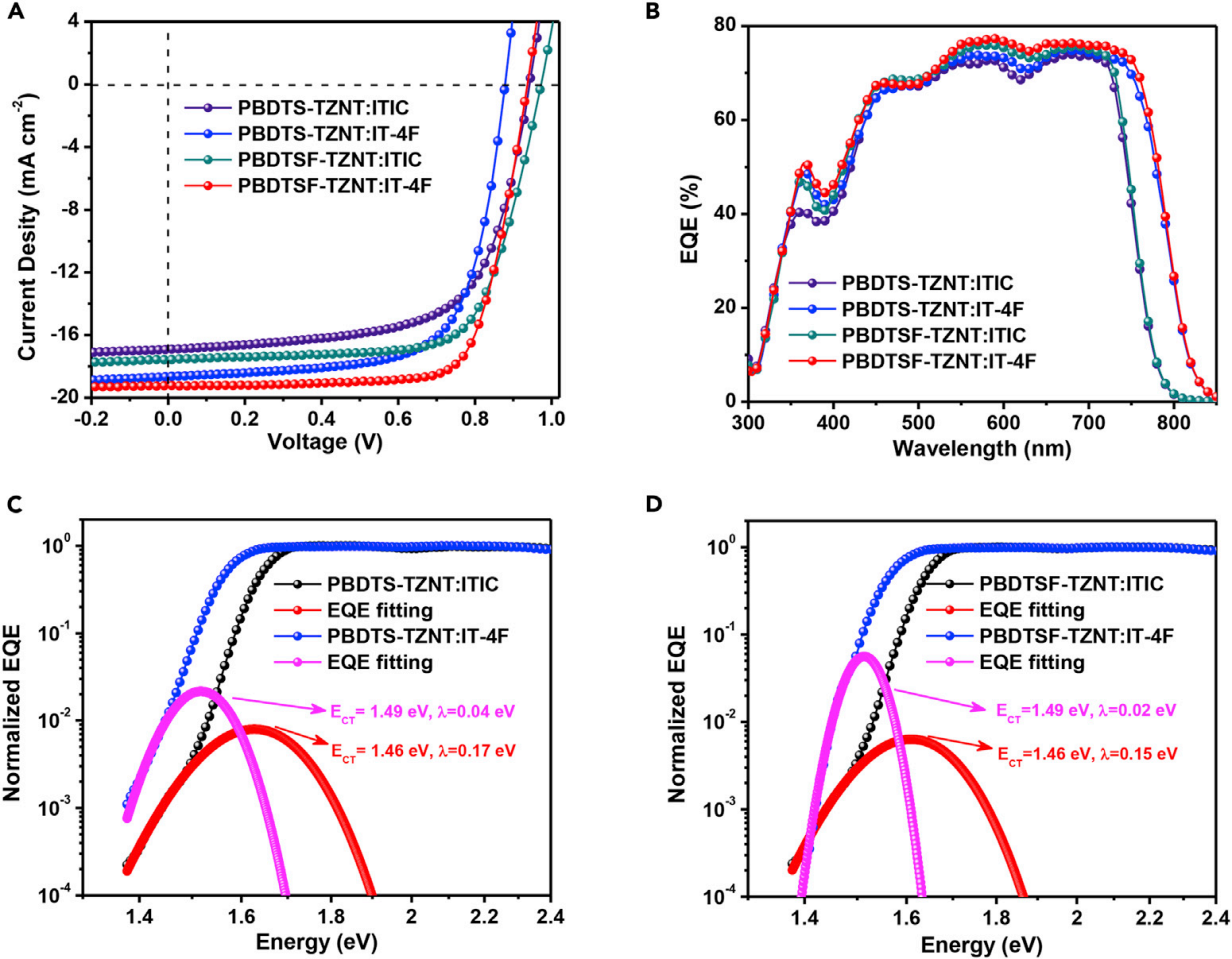
Device Performance of Binary Blend NF-PSCs (A) *J-V* curves of the binary blend NF-PSCs. (B) EQE curves of the binary blend NF-PSCs. (C) The normalized EQE_PV_ spectra in logarithmic scale of PBDTS-TZNT-based binary blend devices. (D) The normalized EQE_PV_ spectra in logarithmic scale of PBDTSF-TZNT-based binary blend devices. Also see Figures S4–S10 and Tables S1–S3.

The photovoltaic properties of the two donor copolymers were investigated by using an inverted device of glass/indium tin oxide (ITO)/ZnO/copolymer:ITIC or IT-4F/MoO_3_/Al. The optimization conditions included the donor/acceptor ratios, solvents, concentrations, solvent additives, and thermal annealing (TA) treatments (Figures S4–S10, Tables S1–S3). The optimized condition was determined to be 1:1 (w/w) with 0.2 v/v% of 1,8-diiodoethane and a total concentration of 14 mg mL^−1^ in CF solution. TA treatment of the as-cast film at 100°C for 5 min before electrode deposition could further optimize blend morphology and improve the device performance. As shown in Figure 3 and Table 2, using ITIC as the acceptor, PBDTS-TZNT-based devices showed a PCE of 10.45% with a *V*_oc_ of 0.94 V, a *J*_sc_ of 16.92 mA cm^−2^, and an FF of 65.7%. A simultaneously improved *V*_oc_ of 0.98 V, *J*_sc_ of 17.57 mA cm^−2^, and FF of 70.6% yielded a higher PCE of 12.16% for PBDTSF-TZNT devices, owing to the lower energy level, higher absorption, and more compact molecular packing of PBDTSF-TZNT. The E_loss_ would be decreased from 0.65 eV for PBDTS-TZNT:ITIC to 0.61 eV for PBDTSF-TZNT:ITIC, which were relatively low among those devices based on BTA-containing donor copolymers. However, the PCEs were still limited by *J*_sc_ and FF, which might be partly due to the limited absorption range and small HOMO offset. Bearing this in mind, IT-4F with a lower HOMO level and red-shifted absorption was selected instead of ITIC. As expected, the slightly decreased *V*_oc_s were observed to be 0.88 for PBDTS-TZNT:IT-4F and 0.93 V for PBDTSF-TZNT:IT-4F devices, respectively. However, the relatively high *V*_oc_ of 0.93 V is still much higher than those of efficient NF-PSCs using IT-4F as the acceptor (Li et al., 2018a, Zhang et al., 2018d, Zheng et al., 2018), thus leading to a very low E_loss_ of 0.59 eV. Nevertheless, such low E_loss_ would not restrict the corresponding charge extraction process. High *J*_sc_ of 19.23 mA cm^−2^ and FF of 74.1% were obtained for PBDTSF-TZNT:IT-4F devices, giving rise to the best PCE of 13.25%. On the other hand, slightly reduced E_loss_ of 0.64 eV and obviously improved PCE of 11.31% could be also achieved in PBDTS-TZNT:IT-4F devices. The above-mentioned device performance improvements were confirmed by the external quantum efficiency (EQE) evaluations (Figure 3B). PSCs based on IT-4F displayed higher and broader photoresponse (300–825 nm) than the ITIC-based devices (300–800 nm). Clearly, PBDTSF-TZNT devices exhibited higher photoresponse than PBDTS-TZNT devices. These were reasonable due to the higher absorptions of IT-4F and PBDTSF-TZNT. The calculated current densities (*J*_EQE_) were 16.36 and 17.22 mA cm^−2^ for PBDTS-TZNT and PBDTSF-TZNT devices using ITIC acceptor, respectively, and these were enhanced to 18.63 and 18.97 mA cm^−2^ if using IT-4F acceptor. The calculated results matched well with those values obtained from *J-V* measurements.

**Table 2:** Photovoltaic Parameters of the NF-PSCs

Active Layer	*V*_oc_ (V)	*J*_sc_ (mA cm^−2^)	*J*_EQE_^b^ (mA cm^−2^)	FF (%)	PCE (%)
PBDTS-TZNT:ITIC	0.94 (0.93 ± 0.01)^a^	16.92 (16.79 ± 0.13)	16.45	65.7 (64.9 ± 0.8)	10.45 (10.13 ± 0.32)
PBDTS-TZNT:IT-4F	0.88 (0.87 ± 0.01)	18.65 (18.54 ± 0.11)	18.23	68.9 (68.2 ± 0.7)	11.31 (11.00 ± 0.31)
PBDTSF-TZNT:ITIC	0.98 (0.97 ± 0.01)	17.58 (17.49 ± 0.09)	17.22	70.6 (69.6 ± 1.0)	12.16 (11.81 ± 0.35)
PBDTSF-TZNT:IT-4F	0.93 (0.92 ± 0.01)	19.23 (19.13 ± 0.10)	18.97	74.1 (73.4 ± 0.7)	13.25 (12.92 ± 0.33)

To gain insight into the low E_loss_ in this work, the charge transfer state energy (E_CT_) and reorganization energy (λ) were explored. E_CT_ and λ were extracted from Equation 1 according to Marcus theory (Marcus, 1989, Zhao et al., 2016).

(Equation 1) $${\text{EQE}}_{PV}\left(E\right)=\frac{f}{E\sqrt{4\pi \lambda kT}}exp\left(\frac{-{\left(E_{CT}+\lambda -E\right)}^{2}}{4\lambda kT}\right)$$

where EQE_PV_ represents the photovoltaic EQE, *f* is proportional to the absorption strength of the charge transfer state and the density of the donor/acceptor interfaces, λ represents the reorganized energy of the charge transfer states, and *E* represents the photon energy. As shown in Figures 3C and 3D, using ITIC as the acceptor, the E_CT_ was 1.46 eV for both devices. Thus the energy losses resulting from charge transfer (E_g_−E_CT_) was 0.13 eV. However, the λ reduced from 0.17 eV (PBDTS-TZNT:ITIC) to 0.15 eV (PBDTSF-TZNT:ITIC). The lower reorganization energy indicated the less energetic disorder of charge transfer states, leading to a smaller voltage deficit in PBDTSF-TZNT:ITIC devices (Xiao et al., 2018). Moreover, using IT-4F as the acceptor, the E_CT_ increased to 1.49 eV for both devices, resulting in the lower E_g_-E_CT_ of 0.03 eV. In the meantime, λ further reduced to 0.04 eV for PBDTS-TZNT:IT-4F and to 0.02 eV for PBDTSF-TZNT:IT-4F devices. Therefore, the lowest E_g_−E_CT_ and λ values of PBDTS-TZNT:IT-4F blend contributed to the smallest E_loss_ in the resulting devices.

**Figure 4:**
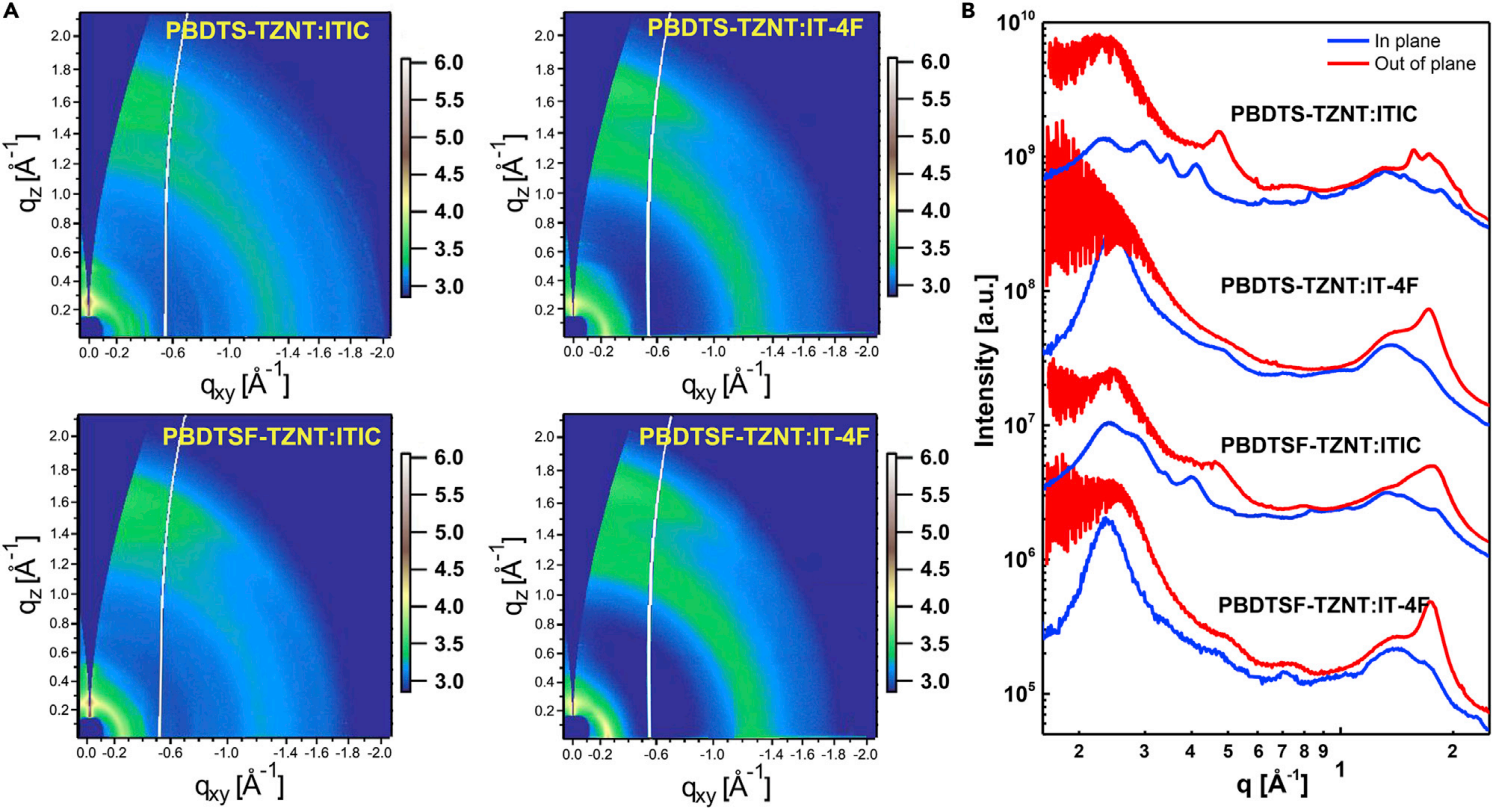
GIWAXS Analysis of Blend Films (A) GIWAXS patterns of PBDTS-TZNT:ITIC, PBDTS-TZNT:IT-4F, PBDTSF-TZNT:ITIC, and PBDTSF-TZNT:IT-4F blend films. (B) In-plane and out-of-plane line-cut profiles of PBDTS-TZNT:ITIC, PBDTS-TZNT:IT-4F, PBDTSF-TZNT:ITIC, and PBDTSF-TZNT:IT-4F blend films. Also see Figure S11 and Table S4.

To gain in-depth structural information on the molecular packing behaviors, 2D grazing incidence wide-angle X-ray scattering was performed (Figures 4 and S11 and Table S4). The neat PBDTS-TZNT and PBDTSF-TZNT films exhibited (100) diffraction peak at q_xy_ = 0.25 Å^−1^, corresponding to their lamellar stacking distance (d_L_) of 25.1 Å, which was due to their identical alkyl side chains. However, PBDTSF-TZNT film displayed a smaller π-π stacking distance (d_π_) of 3.61 Å (q_z_ = 1.74 Å^−1^) and a larger coherent length (L_C_) of 25 Å than PBDTS-TZNT (3.63 Å, q_z_ = 1.73 Å^−1^, L_C_ = 23 Å). The results indicated that the more ordered molecular packing of PBDTSF-TZNT was formed, owing to the introduced noncovalent effect by fluorination. After blending with ITIC, their high crystallinities were lowered to some extent, along with the reduced L_C_s of 16 and 22 Å for PBDTS-TZNT:ITIC and PBDTSF-TZNT:ITIC, respectively. The PBDTS-TZNT:ITIC blend exhibited much stronger (100) diffraction along the q_z_ direction than the q_xy_ direction, indicating its more preferred edge-on packing. However, a more balanced coexistence of face-on and edge-on packing of PBDTSF-TZNT:ITIC was observed to enable the more efficient three-dimensional charge transfer. If using IT-4F instead of ITIC, significantly improved diffractions were observed with the increased L_C_s of 26 and 30 Å for PBDTS-TZNT:IT-4F and PBDTSF-TZNT:IT-4F, respectively, showing their enhanced crystallinities. Moreover, both blends showed stronger (100) diffraction along the q_xy_ direction than the q_z_ directions, indicating that more preferred face-on packing was realized. Such stacking change would be positive for vertical charge transfer in PSC devices. The hole mobility (*μ*_h_) and electron mobility (*μ*_e_) of the blend films were estimated by space-charge-limited current method (Figure S12). The *μ*_h_/*μ*_e_ of PBDTS-TZNT:ITIC, PBDTS-TZNT:IT-4F, PBDTSF-TZNT:ITIC, and PBDTS-TZNT:IT-4F blends were measured to be 1.04 × 10^−4^/3.26 × 10^−5^, 2.65 × 10^−4^/1.26 × 10^−4^, 3.21 × 10^−4^/1.87 × 10^−4^, and 5.10 × 10^−4^/4.03 × 10^−4^ cm^2^ V^−1^ s^−1^, respectively. As could be concluded, the improved crystallinity promoted by fluorination on the conjugated side chains of donor polymer and the end group of the acceptor would benefit the charge carrier mobilities. Finally, the highest and most balanced *μ*_h_/*μ*_e_ ratio of PBDTS-TZNT:IT-4F correlated to the highest *J*_sc_ and FF values in the related NF-PSCs.

**Figure 5:**
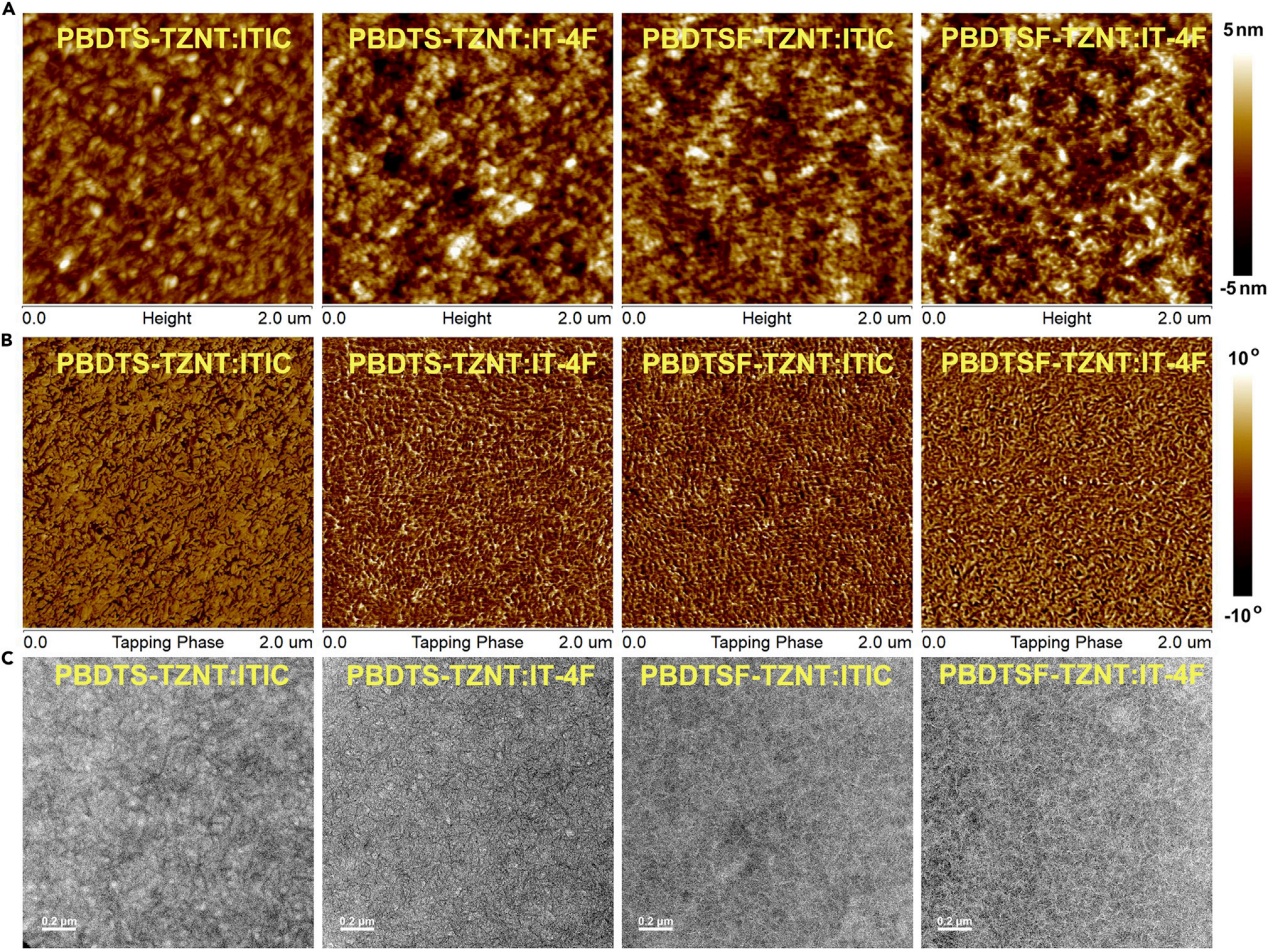
Analysis of the Morphology of Blend Films (A) Atomic force microscopy (AFM) height images of PBDTS-TZNT:ITIC, PBDTS-TZNT:IT-4F, PBDTSF-TZNT:ITIC, and PBDTSF-TZNT:IT-4F blend films. (B) AFM phase images of PBDTS-TZNT:ITIC, PBDTS-TZNT:IT-4F, PBDTSF-TZNT:ITIC, and PBDTSF-TZNT:IT-4F blend films. (C) Transmission electron microscopic (images) of PBDTS-TZNT:ITIC, PBDTS-TZNT:IT-4F, PBDTSF-TZNT:ITIC, and PBDTSF-TZNT:IT-4F blend films. Also see Figure S13.

Resonant soft X-ray scattering (RSoXS) experiments were carried out to study the nanophase separation and the average composition distribution of the blend films (Figure S13). The PBDTS-TZNT:ITIC blend exhibited relatively low scattering intensity with two peaks located at 0.03 and 0.12 nm^−1^, respectively, corresponding to the large domain sizes of 105 and 26 nm, showing the non-uniform nanophase separation. Higher scattering intensity with slightly reduced domain size of 24 nm was observed for the PBDTSF-TZNT:ITIC blend, showing the improved domain purity and better morphology. If replacing ITIC with IT-4F, both polymer blends exhibited significantly improved scattering intensity with further reduced domain sizes of 24 nm for PBDTS-TZNT:IT-4F and 18 nm for PBDTSF-TZNT:IT-4F. This was due to the faster crystallization of IT-4F-based blends during the film deposition. The phase separation could be visually observed by atomic force microscopy and transmission electron microscopy (Figure 5). PBDTS-TZNT:ITIC appeared to have a fairly smooth top surface with a root-mean-square (RMS) roughness of 0.78 nm. The coexistence of large and small domains could be observed, which agreed well with the RSoXS results. Gradually increasing RMS values of 1.14, 1.45, and 1.53 nm with more fibril-like interpenetrating networks could be observed in PBDTSF-TZNT:ITIC and PBDTS-TZNT:IT-4F blends. The better nanofibril domains in PBDTSF-TZNT:IT-4F with the highest domain purity provided the most efficient exciton dissociation and charge transfer for realizing the best device performance.

**Figure 6:**
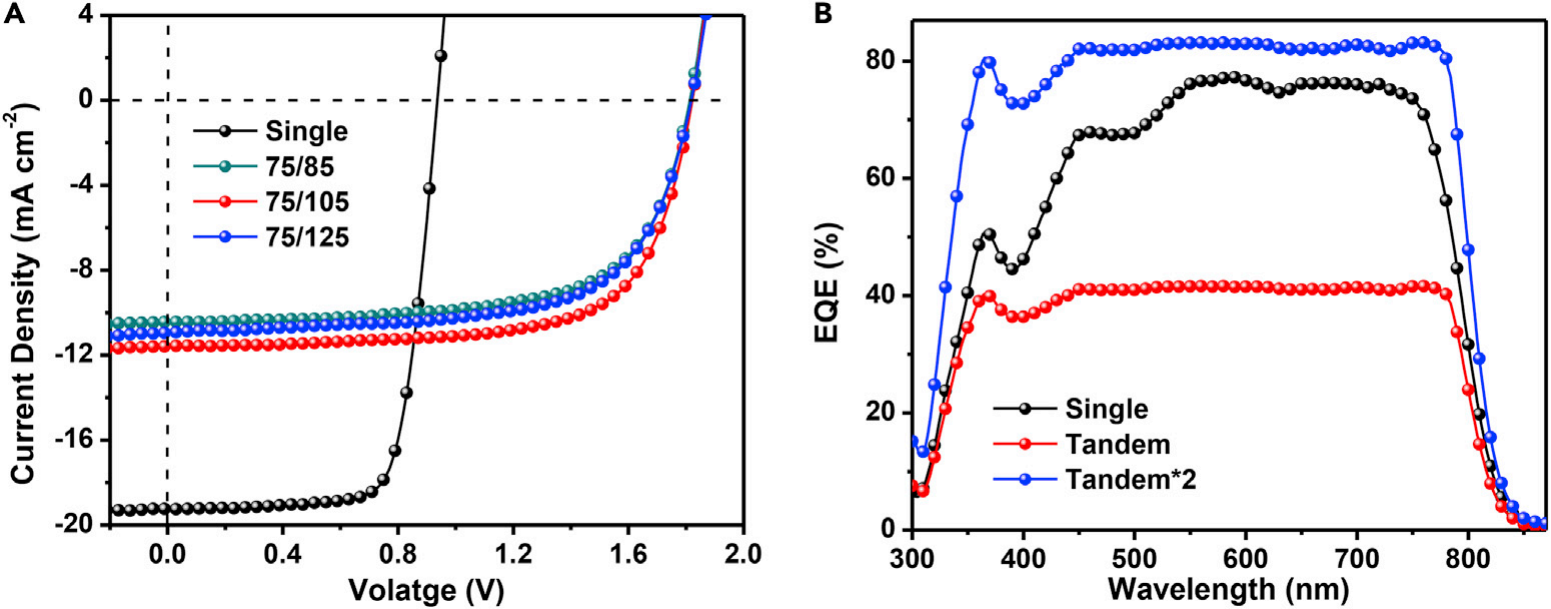
Device Performance of Homo-Tandem Cells (A) *J-V* curves of the single-junction and homo-tandem devices based on PBDTSF-TZNT:IT-4F blend layer. (B) EQE curves of the single-junction and homo-tandem devices based on PBDTSF-TZNT:IT-4F blend layer. Also see Figure S14.

Although high PCEs had been achieved in single-junction NF-PSCs, the device performance was still limited by incomplete light harvesting. To make full use of sunlight and further increase the device performance, homo-tandem cells were fabricated and evaluated with the device configuration of glass/ITO/ZnO/PBDTSF-TZNT:IT-4F/PEDOT:PSS/ultrathin Ag/ZnO/PBDTSF- TZNT:IT-4F/MoO_3_/Ag. To get enough high PCE, the active layer thickness of the bottom cell was determined to be 75 nm and thickness of the top cell ranged from 85 to 125 nm (Figure 6 and Table 3). When the thickness of the top cell was 105 nm, the resulting devices exhibited the best PCE of 14.52% with a *V*_oc_ of 1.82 V, a *J*_sc_ of 11.58 mA cm^−2^, and an FF of 68.9%. The device performance was certified by a third party as a PCE of 14.14% (Figure S14). To the best of our knowledge, this is a new record for homo-tandem PSCs. On the other hand, over 80% of the original device performance could be retained after storing in the glove box for 300 h (Figure S15). The EQE spectra confirmed the improved light-harvesting ability (Figure 6B). The EQE of the homo-tandem device here is defined as the ratio of the total converted carriers by the two subcells to the sum of the incident photons and is estimated by measuring the photoresponse of the tandem cell and then multiplying it by 2 to represent the total number of photons being converted to electrons (Xu et al., 2018b). The fabricated homo-tandem devices displayed high EQE response of around 40% from 450 to 750 nm with a highest value of 41.5%. Thus the total EQE response of the homo-tandem solar cells reached 83% on superposition of two subcells. The calculated *J*_EQE_ was 11.27 mA cm^−2^, which agreed well with the *J-V* result.

**Table 3:** Photovoltaic Parameters of the Homo-Tandem NF-PSCs

Film Thickness (nm)	*V*_oc_ (V)	*J*_sc_ (mA cm^−2^)	FF (%)	PCE (%)
75/85	1.81 (1.79 ± 0.02)	10.43 (10.28 ± 0.15)	66.7 (65.4 ± 1.3)	12.59 (12.04 ± 0.55)
75/105	1.82 (1.80 ± 0.02)	11.58 (11.47 ± 0.11)	68.9 (67.9 ± 1.0)	14.52 (14.02 ± 0.50)
75/125	1.81 (1.79 ± 0.02)	10.95 (10.81 ± 0.14)	65.9 (64.5 ± 1.4)	13.06 (12.48 ± 0.58)

The averaged photovoltaic parameters in parentheses were obtained from at least 15 devices.

Also see Figure S15.

### Conclusions

Two novel TZNT-containing WBG polymers, PBDTS-TZNT and PBDTSF-TZNT, were successfully designed and synthesized for highly efficient NF-PSCs with low energy loss. The rigid planar backbone of BDT and TZNT units provided these copolymers with high crystallinity and good molecular packing. Sulfuration and fluorination side chains of BDT lowered the HOMO level (5.39 eV for PBDTS-TZNT and −5.45 eV for PBDTSF-TZNT) for guaranteeing high *V*_oc_ up to 0.98 V using ITIC as the acceptor. If selecting IT-4F instead of ITIC, the relatively low E_loss_ of 0.59 eV would be realized even keeping the high *V*_oc_ of 0.93 V. Beside this, broadened absorption, better matched energy level, further improved crystallinity, and fine-tuned morphology enabled PBDTSF-TZNT-based devices to promote the PCE from 12.16% to 13.25%. Moreover, to make full use of sunlight, homo-tandem devices based on PBDTSF-TZNT:IT-4F were fabricated and evaluated, which exhibited the highest PCE of 14.52% with a *V*_oc_ of 1.82 V, a *J*_sc_ of 11.58 mA cm^−2^, and an FF of 68.9%. This work demonstrated that fine-tuning the electronic energy level and molecular packing of WBG donor copolymers and pairing them with a proper LBG acceptor is a promising way to realize high efficiency and low energy loss at the same time in NF-PSCs.

## Methods

All methods can be found in the accompanying Transparent Methods supplemental file.

## Supplemental Information

Document S1. Transparent Methods, Figures S1–S15, and Tables S1–S4
