# Supplementary material for: Low-Energy-Loss Polymer Solar Cells with 14.52% Efficiency Enabled by Wide-Band-Gap Copolymers
Source: iScience. 2019 Jan 6;12:1–12. doi: 10.1016/j.isci.2018.12.027 (PMC6348165; doi:10.1016/j.isci.2018.12.027)
Supplement: Document S1. Transparent Methods, Figures S1–S15, and Tables S1–S4 [file mmc1.pdf]

ISCI, Volume *12*

Supplemental Information

Low-Energy-Loss Polymer Solar Cells with 14.52% Efficiency Enabled by Wide-Band-Gap Copolymers

Kui Feng, Jian Yuan, Zhaozhao Bi, Wei Ma, Xiaopeng Xu, Guangjun Zhang, and Qiang Peng

Transparent Methods

Materials and Characterization: ITIC and IT-4F were purchased from Derthon Optoelectronic Materials Science Technology. The organic tin monomer (M2 and M3) and naphtha[1,2-c:5,6-c]bis(1H-[1,2,3]triazole) (compound 1) were synthesized according to previous procedures (Dong et al. 2013; Zhang et al. 2018). All the other chemicals were purchased from Aladdin, Adamas, Sigma-Aldrich, and Alfa Asear Chemical Co., and used without further purification. All solvents were freshly distilled immediately prior to use.

Synthesis of Compound 2: Compound 1 (1.00 g, 4.78 mmol) was dissolved in the dimethyl sulfoxide (DMSO, 50 mL) at the room temperature. Sodium hydroxide (1.0 g, 25 mmol), tetrabutylammonium bromide (TBAB 0.1 g, 0.31 mmol) and 2-butyloctyl bromide (0.47 g, 0.19 mmol) were added successively under the protection of argon. The reaction was stirred under room temperature for 24 h. After that, the mixture was poured into ice water. The organic layer was extracted with dichloromethane and washed for several times with H_2O. The solution was dried over anhydrous $MgSO_4$ and concentrated by rotary evaporation. The residue was purified by column chromatography (SiO_2, petroleum ether:dichloromethane = 1:2) to give compound 2 as a colorless liquid (0.39 g, yield: 15.1%). ^{1}H NMR (400 MHz, $CDCl_3$, δ/ppm): 8.50-8.48 (d, 2H, J = 9.0 Hz, ArH), 8.00-7.99 (d, 2H, J = 9.0 Hz, ArH), 4.70-4.68 (d, 4H, J=7.1 Hz, N-CH_2), 2.39-2.38 (m, 2H, CH), 1.38-1.24 ((m, 32H, CH_2), 0.90-0.86 (t, 12H, CH_3). ^{13}C NMR (100 MHz, $CDCl_3$, δ/ppm): 142.9, 142.2, 122.6, 122.5, 117.3, 60.2, 39.2, 31.7, 31.4, 31.1, 29.7, 29.5, 28.4, 26.2, 22.9, 22.6,

14.1, 14.0. Anal. Calcd for $C_{34}H_{54}N_6$ (%): C 74.68, H 9.96, N 15.37; found (%): C 75.98, H 9.76, N 15.22.

Synthesis of Compound 3: After compound 2 (0.62 g, 1.15 mmol) was added in hydrobromic acid (20 mL), the temperature was raised until the solid was dissolved completely. Bromine (0.40 g, 2.53 mmol) was then added dropwise and the reaction was stirred overnight under reflux. After that, the excessive solution of sodium bisulfite was added to remove the excess bromine. The crude product was poured into 200 mL water and extracted by dichloromethane for three times. The solution was dried over anhydrous $MgSO_4$ and concentrated by rotary evaporation. The residue was purified by column chromatography (SiO_2, petroleum ether:dichloromethane = 1:1) to give compound 3 as a colorless liquid (0.54 g, yield: 68.2%). ^{1}H NMR (400 MHz, $CDCl_3$, δ/ppm): 8.64 (s, 2H, ArH), 4.72-4.71 (d, 4H, J=7.1 Hz, N-CH_2), 2.37-2.35 (m, 2H, CH), 1.38-1.25 ((m, 32H, CH_2), 0.89-0.86 (t, 12H, CH_3). ^{13}C NMR (100 MHz, $CDCl_3$, δ/ppm): 142.4, 141.1, 124.6, 122.3, 110.4, 60.7, 39.1, 31.7, 31.2, 31.0, 29.7, 29.5, 28.4, 26.1, 22.9, 14.1, 13.9. Anal. Calcd for $C_{34}H_{52}N_6Br_2$(%): C 57.95, H 7.44, N 11.93; found (%): C 75.98, H 7.76, N 15.53.

Synthesis of Compound 4: Compound 3 (0.5 g, 0.71 mmol), tributyl-(2-thienyl)-stannane (0.53 g, 1.42 mmol) and $Pd(PPh_3)_4$ (10 mg) were added in toluene. The mixture was stirred under the protection of argon and refluxed for 6 h. After the evaporation of the solvent, the product was purified by silica column chromatography (SiO_2, petroleum ether:dichloromethane = 1:1) to give compound 4 as an orange solid (0.41 g, yield: 81.2%). ^{1}H NMR(400 MHz, $CDCl_3$, δ/ppm): 8.69 (s,

2H, ArH), 8.27-8.26 (d, 2H, *J*=7.1 Hz, ArH), 7.45-7.43 (d, 2H, *J*=7.1 Hz, ArH), 7.24-7.23 (m, 2H, ArH), 4.77-4.76 (d, 4H, *J*=9.1 Hz, N-CH_2), 2.39-2.36 (m, 2H, CH), 1.38-1.29 (m, 32H, CH_2), 0.92-0.88 (t, 12H, CH_3). ^{13}C NMR (100 MHz, $CDCl_3$, δ/ppm): 140.1, 128.04, 127.7, 126.0, 123.9, 122.0, 118.0, 60.1, 39.2, 31.8, 31.4, 31.1, 29.6, 28.5, 26.2, 22.9, 22.67, 14.2, 14.0. Anal. Calcd for $C_{42}H_{58}N_6$ S_2 (%): C 70.94, H 8.22, N 11.82; found (%): C 70.65, H 7.96, N 11.54.

Synthesis of Compound M1: Compound 4 (0.61 g, 0.857 mmol) was dissolved in THF (30 mL) and then N-bromosuccinimide (NBS) (0.33 g, 1.88 mmol) was added in several portions. The mixture was reacted for 8 h at the room temperature. After the evaporation of the solvent, the product was purified by silica column chromatography (SiO_2, petroleum ether:dichloromethane = 3:1) to give compound M1 as an orange solid (0.60 g, yield: 80.4%). ^{1}H NMR (400 MHz, $CDCl_3$, δ/ppm): 8.58 (s, 2H, ArH), 7.97-7.95 (d, 2H, *J*=3.9 Hz, ArH), 7.17-7.15 (d, 2H, *J*=3.9 Hz, ArH), 4.75-4.74 (d, 4H, *J*=6.8 Hz, N-CH_2), 2.39-2.36 (m, 2H, CH), 1.38-1.29 ((m, 32H, CH_2), 0.92-0.88 (t, 12H, CH_3). ^{13}C NMR (100 MHz, $CDCl_3$, δ/ppm): 141.4, 130.8, 127.0, 117.6, 113.7, 60.1, 39.2, 31.9, 31.7, 31.41, 31.1, 29.7, 29.5, 29.3, 28.4, 26.1, 22.9, 22.6, 14.0, 14.0, 1.03. Anal. Calcd for $C_{42}H_{56}N_6Br_2S_2$(%): C 58.06, H 6.50, N 9.67; found (%): C 58.35, H 6.46, N 9.87.

Synthesis of PBDTS-TZNT: M2 (0.350 g, 0.262 mmol) and M1 (0.227 g, 0.262 mmol) were first dissolved in 10 mL degassed toluene. After that, $Pd_2(dba)_3$ (4.7 mg, 2% mmol) and P(o-tol)$_3$ (6.3 mg, 8% mmol) were added into the above solution under argon atmosphere. The mixture was then stirred at 110 °C for 24 h in dark. After the

reaction mixture was cooled down to room temperature, it was dropped into 500 mL methanol. The resulting solid was collected by filtration, and then subjected to Soxhlet extraction successively with methanol, acetone, and hexane to remove the oligomers and impurities. The remaining polymer was dissolved in chloroform and precipitated again from methanol to yield PBDTS-TZNT as a dark solid (0.21 g, yield: 63.3%). ^{1}H NMR (400 MHz, $CDCl_3$, δ/ppm): 8.65-8.44 (br, 2H, ArH), 8.41-7.44 (br, 4H, ArH), 7.25-7.19 (br, 6H, ArH), 4.79-4.77 (br, 5H, N-CH_2), 3.12-3.10 (br, 4H, S-CH_2), 2.39-2.36 (m, 2H, CH), 1.52-1.19 (br, 64H, CH_2), 0.89-0.85 (br, 24H, CH_3). Anal. Calcd for $(C_{84}H_{112}N_6S_8)_n$ (%): C 68.99, H 7.72, N 5.75; found: C 68.89, H 7.51, N 5.61.

Synthesis of PBDTSF-TZNT: M3 (0.320 g, 0.233 mmol) and M1 (0.233 g, 0.233 mmol) were dissolved in 10 mL degassed toluene. After that, $Pd_2(dba)_3$ (4.2 mg, 2% mmol) and $P(o\text{-}tol)_3$ (5.7 mg, 8% mmol) were added into the above solution under argon atmosphere. The mixture was then stirred at 110 °C for 24 h in dark. After the reaction mixture was cooled down to room temperature, it was dropped into 500 mL methanol. The resulting solid was collected by filtration, and then subjected to Soxhlet extraction successively with methanol, acetone, and hexane to remove the oligomers and impurities. The remaining polymer was dissolved in chloroform and precipitated again from methanol to yield PBDTS-TZNT as a dark solid (0.20 g, yield: 60.1%). ^{1}H NMR (400 MHz, $CDCl_3$, δ/ppm): 8.65-8.40 (br, 2H, ArH), 8.39-7.40 (br, 2H, ArH), 7.25-7.19 (br, 6H, ArH), 4.82-4.80 (br, 5H, N-CH_2), 3.14-3.12 (br, 4H, S-CH_2), 2.50-2.42 (m, 2H, CH), 1.68-1.19 (br, 64H, CH_2),

0.89-0.85 (br, 24H, CH_3). Anal. Calcd for $(C_{84}H_{110}F_2N_6S_8)_n$ (%): C 67.33, H 7.40, N 5.61; found: C 67.54, H 7.32, N 5.85.

Instruments and characterization: ^{1}H and ^{13}C NMR spectra were recorded on a Bruker Avance-400 spectrometer with *d*-chloroform as solvent and chemical shifts were reported as δ value (ppm) relative to an internal tetramethylsilane (TMS) standard. The elemental analysis was performed on a Thermo Electron SPA Flash EA 1112 series analyzer. Molecular weights of the copolymers were determined by using a Waters 1515 GPC instrument with THF as the eluent and polystyrene as a standard. Thermogravimetric analysis was conducted on a TA Instrument Model SDT Q600 simultaneous TGA/DSC analyzer at a heating rate of 10 ^{o}C min^{-1} and under a N_2 flow rate of 90 mL min^{-1}. UV-vis spectra were obtained on a Cary 300 spectrophotometer. Cyclic voltammetry measurements were made on a CHI660 potentiostat/galvanostat electrochemical workstation at a scan rate of 50 mV s^{-1}, with a platinum wire counter electrode and an Ag/AgCl reference electrode in an anhydrous and nitrogen-saturated 0.1 mol L^{-1} acetonitrile solution of tetrabutylammonium perchlorate. The $CHCl_3$ solutions of the polymers were drop-coated onto the platinum plate working electrodes. 2D-GIWAXS measurements were performed at beamline 7.3.3 8 at the Advanced Light Source (ALS). R-SoXS transmission measurements were performed at beamline 11.0.1.2 at the ALS. AFM images were obtained by using a Bruker Inova atomic microscope in tapping mode. TEM images were obtained by using a ZEISS LIBRA 200 FE transmission electron microscope. SCLC is described by $J=9\varepsilon_0\varepsilon_r\mu V^2/8L^3$, where J is the current density, L is the film thickness of the active

layer, μ is the hole or electron mobility, ε_r is the relative dielectric constant of the transport medium, ε_0 is the permittivity of free space (8.85×10^{-12} F m^{-1}), V is the internal voltage in the device and $V = V_{appl} - V_{bi} - V_a$, where V_{appl} is the applied voltage to the device, V_{bi} is the built-in voltage due to the relative work function difference of the two electrodes and V_a is the voltage drop due to contact resistance and series resistance across the electrodes.

Single-junction device fabrication: The device structure was ITO/ZnO/copolymer:acceptor/MoO_3/Ag. The pre-patterned ITO glass substrates (sheet resistance = 15 Ω sq^{-1}) were ultrasonicated in detergent, deionized water, acetone, and isopropanol, subsequently. After dried by high-pressure air flow, the substrates were further cleaned by UV-ozone exposure for 30 min. A thin layer (30 nm) of ZnO was formed by spin-coating the precursor solution (diethylzinc solution 2M in toluene, diluted with THF) at 5000 rpm for 30 s, and then baked at 150 °C for about 20 min. The blend films of copolymer:acceptor (1:1, w/w) were prepared by spin-coating their solutions in CF:DIO (99.8:0.2, v/v) mixed solvent (total concentration: 14 mg mL^{-1}). The final film thickness was around 100 nm, detected by a Dektak 6 M surface profilometer. Thin layers of MoO_3 (10 nm) and Ag (100 nm) were then deposited on the top of surface in a high vacuum chamber (2×10^{-4} Pa). The device area was 4.0 mm^2.

Homo-tandem device fabrication: The homo-tandem device structure was ITO/ZnO/PBDTSF-TZNT:IT-4F/PEDOT:PSS/ultrathin Ag/ZnO/PBDTSF-TZNT:IT-4F/PEDOT:PSS/MoO_3/Ag. The fabrication of the bottom sub-cell was similar to

the single-junction device with the film thickness of 75 nm to guarantee sufficient light harvesting in the top sub-cell. Poly(3,4-ethylenedioxythiophene):polystyrene sulfonate (PEDOT:PSS) with 0.5 v% of Triton X-100 was spin-coated on the top of active layer of bottom cell (40 nm), followed by thermal evaporation of ultrathin Ag layer (5 nm) and ZnO nanoparticle layer spin-coating (30 nm), which formed the recombination layer. ZnO nanoparticles were synthesized by the previous literature (Beek et al. 2005). The active layer film thickness of the top cell varied from 85 to 125 nm to obtain a balanced current output in both sub-cells. Finally, thin layers of MoO_3 (10 nm) and Ag (100 nm) were deposited in a high vacuum chamber (2×10^{-4} Pa).

Device characterization: The I-V characterization of the devices was carried out on a computer-controlled Keithley 2400 Source Measurement system under 100 mW/cm^{-2} AM 1.5G light source, provided by a AAA solar simulator (XES-70S1, SAN-EI Electric Co., Ltd), which was calibrated with a standard Si solar cell (AK-200, KONICA MINOLTA, INC.). The EQE values were measured with a Newport QE test system (Newport Co., Ltd.) during illumination with monochromatic light from a xenon lamp, monitored by a traceable silicon photodiode.

Supplemental Figures

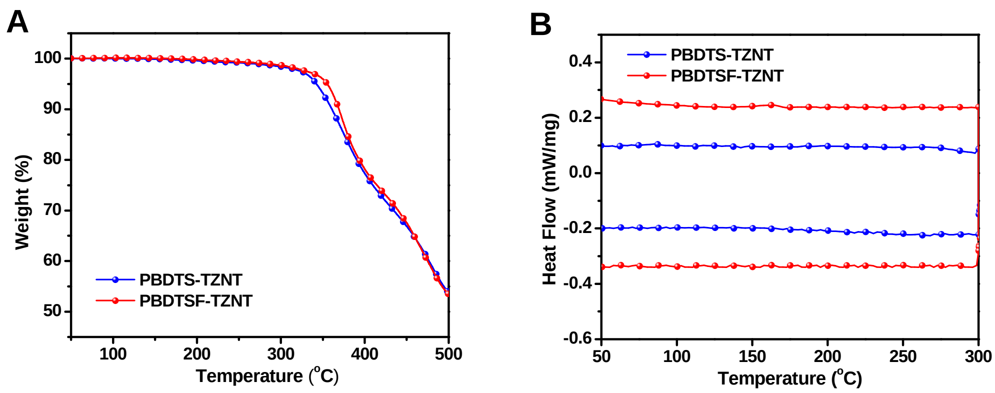

Figure S1. (**A**) TGA curves of the copolymers. (**B**) DSC curves of the copolymers. Related to Figure 1 and Scheme 1.

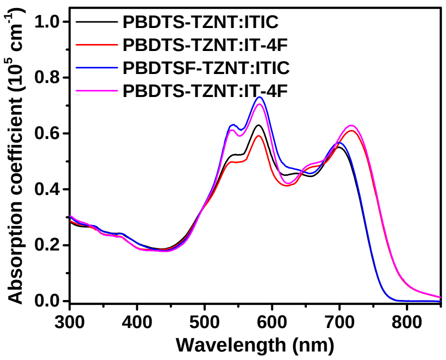

Figure S2. UV-vis spectra of the blend films. Related to Figure 2 and Table 1.

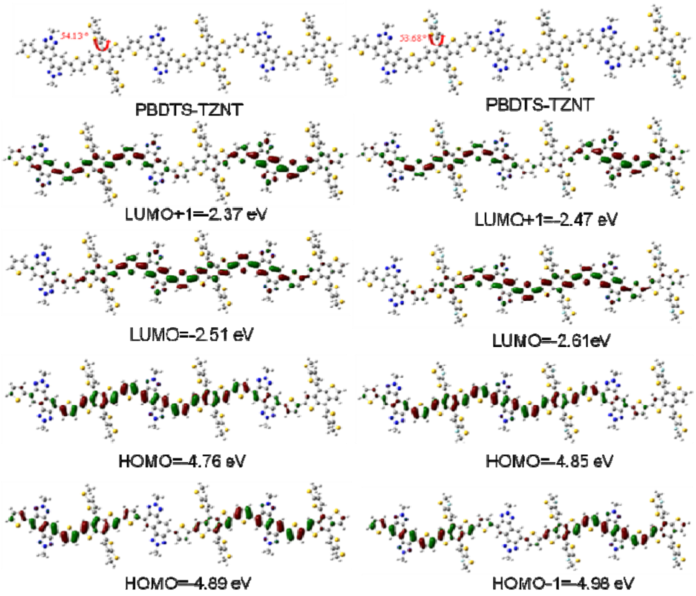

Figure S3. Optimized geometries of polymer trimers and the simulated energy levels**.** Related to Figure 2.

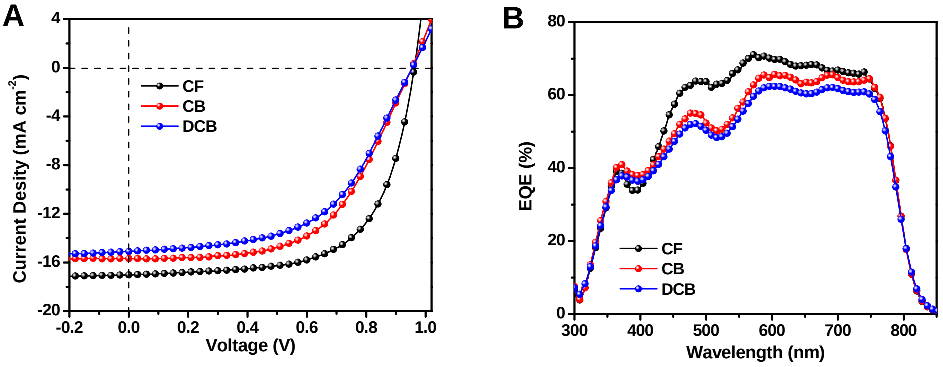

Figure S4. J-V curves (**A**) and EQE curves (**B**) of the PBDTSF-TZNT:IT-4F (1:1, w/w) devices processed from different solvents. Related to Figure 3 and Table 2.

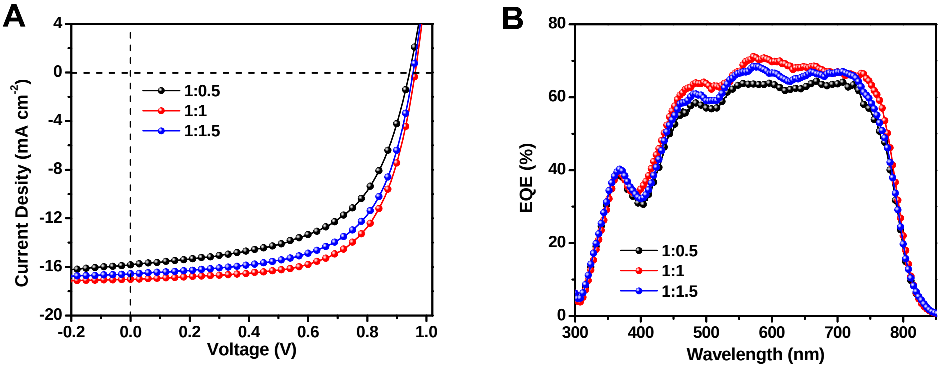

Figure S5. J-V curves (**A**) and EQE curves (**B**) of the PBDTSF-TZNT:IT-4F devices processed from CF with different D/A ratios. Related to Figure 3 and Table 2.

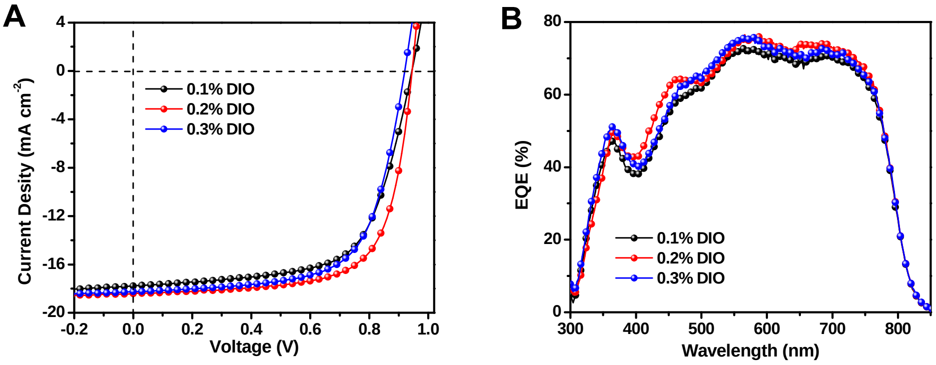

Figure S6. *J-V* curves (**A**) and EQE curves (**B**) of the PBDTSF-TZNT:IT-4F devices processed from CF with different additive content. Related to Figure 3 and Table 2.

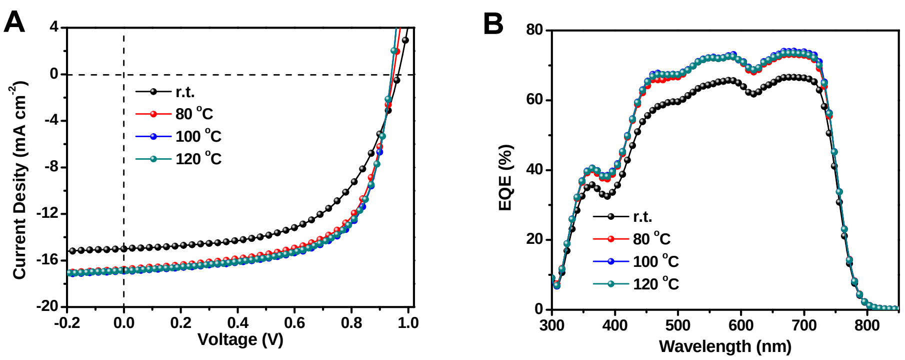

Figure S7. *J-V* curves (**A**) and EQE curves (**B**) of the PBDTS-TZNT:ITIC devices with different thermal annealing temperatures. Related to Figure 3 and Table 2.

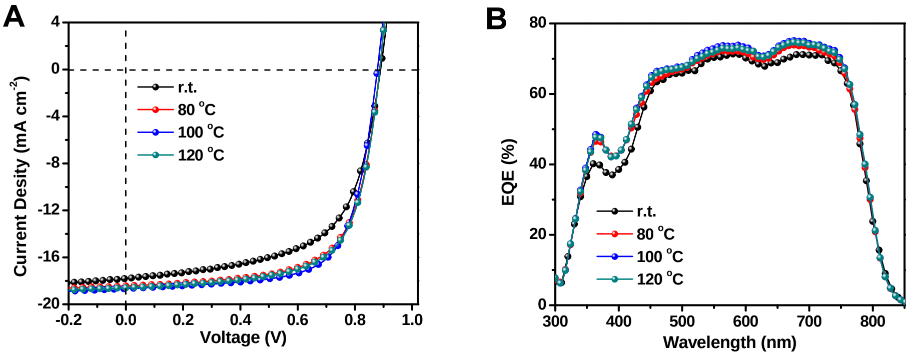

Figure S8. *J-V* curves (**A**) and EQE curves (**B**) of the PBDTS-TZNT:IT-4F devices with different thermal annealing temperatures. Related to Figure 3 and Table 2.

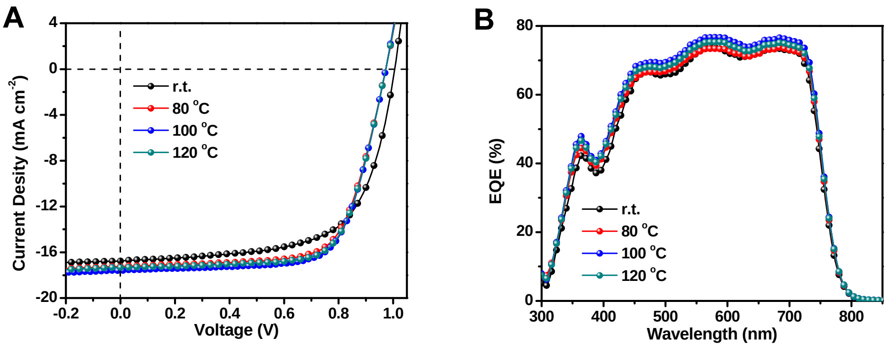

Figure S9. *J-V* curves (**A**) and EQE curves (**B**) of the PBDTSF-TZNT:ITIC devices with different thermal annealing temperatures. Related to Figure 3 and Table 2.

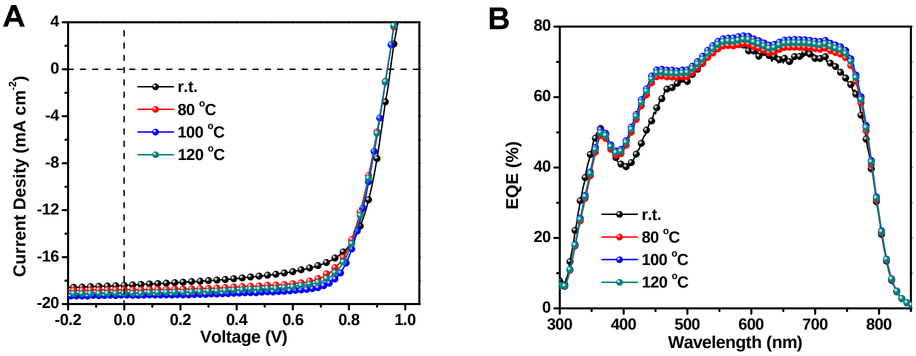

Figure S10. *J-V* curves (**A**) and EQE curves (**B**) of the PBDTSF-TZNT:IT-4F devices with different thermal annealing temperatures. Related to Figure 3 and Table 2.

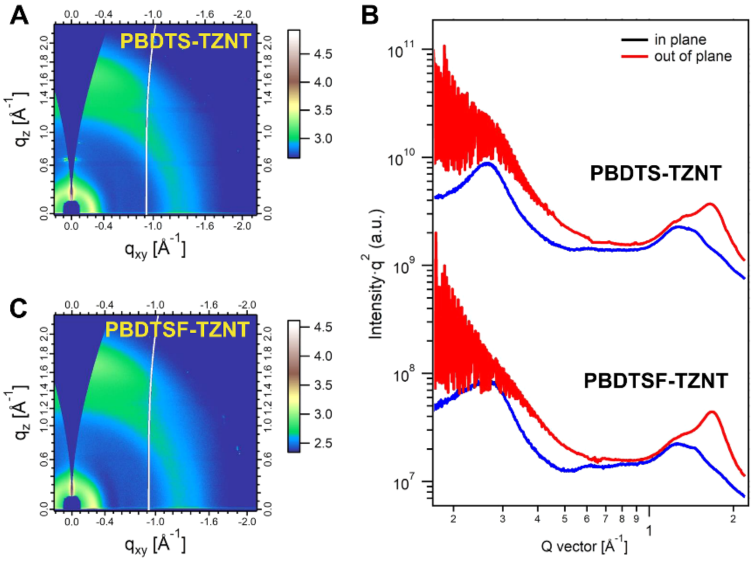

Figure S11. (**A,B**) GIWAXS patterns of pristine polymer films. (**C**) In-plane and out-of-plane line-cut profiles. Related to Figure 4.

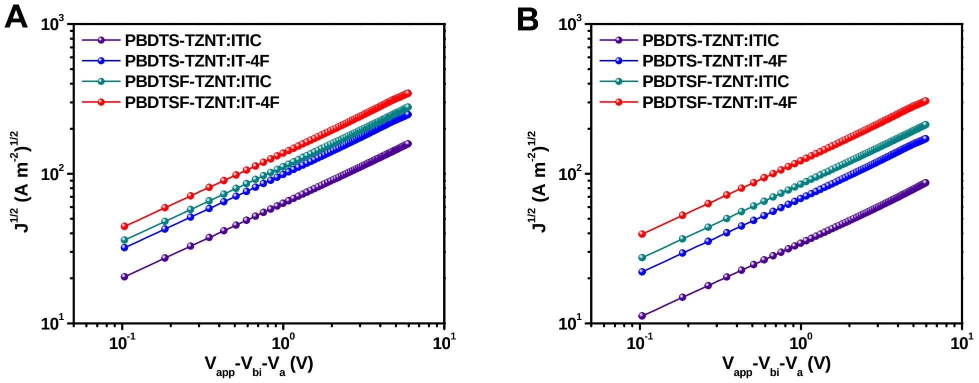

Figure S12. $J^{1/2}$-V curves of the hole-only (**A**) and electron-only (**B**) devices. Related to Figure 4.

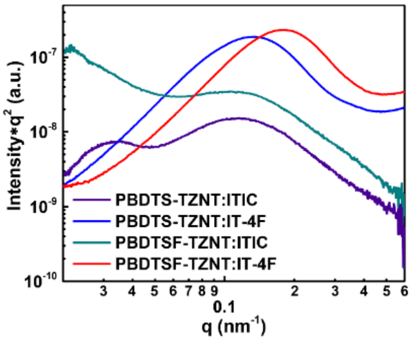

Figure S13. R-SoXS profiles of the blend films. Related to Figure 5.

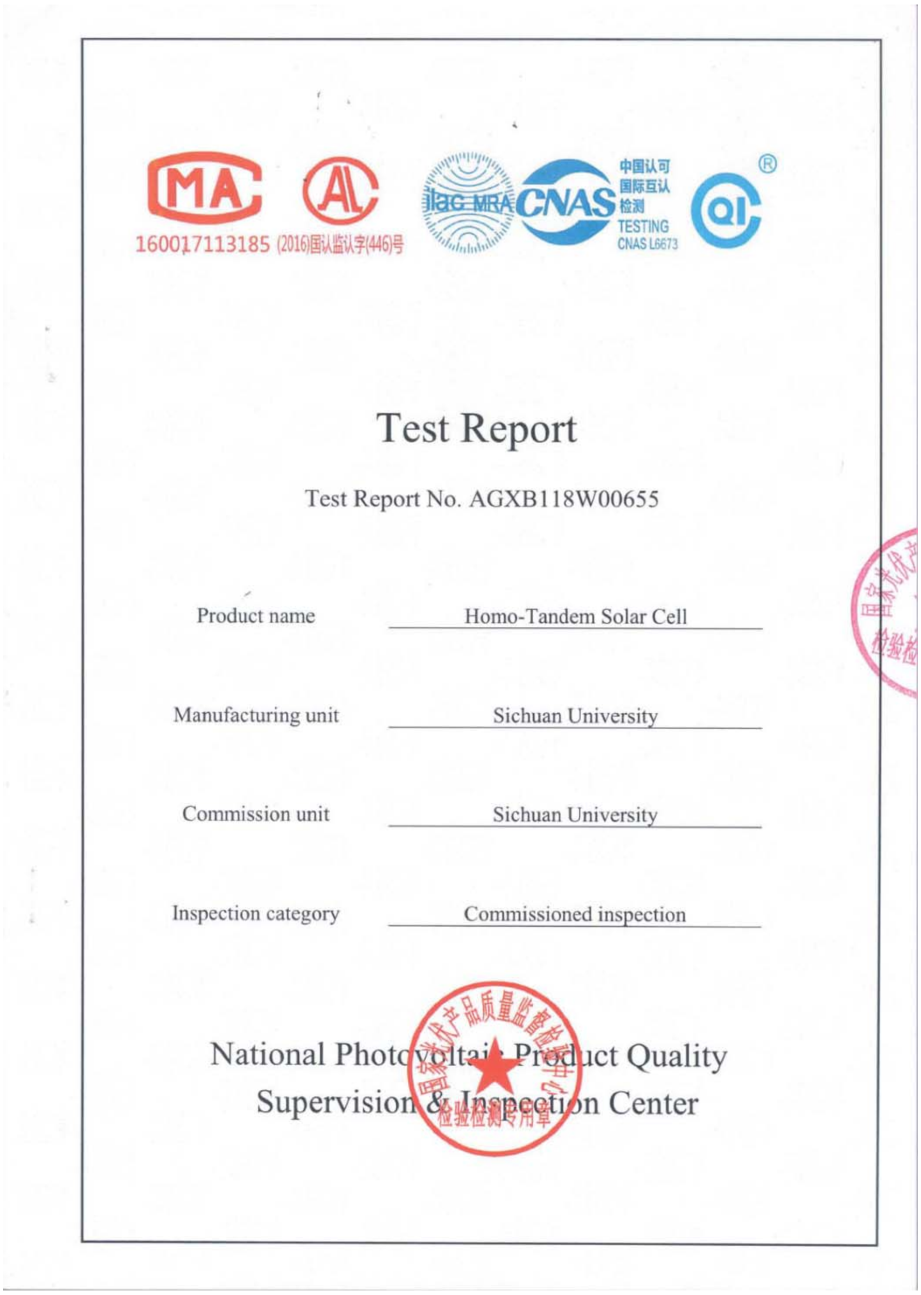

160017113185 (2016)国认监认字(446)号

中国认可
国际互认
检测
TESTING
CNAS L6673

Test Report

Test Report No. AGXB118W00655

Product name	Homo-Tandem Solar Cell
Manufacturing unit	Sichuan University
Commission unit	Sichuan University
Inspection category	Commissioned inspection

National Photovoltaic Product Quality Supervision & Inspection Center

National Photovoltaic Product Quality Supervision & Inspection Center
TEST REPORT

Test Report No. AGXB118W00655

Product name	Homo-Tandem Solar Cell	Trade Mark	/
Date of manufacture	11/26/2018	Model / type	/
Sample No.	1#	Sample grade	Qualified product
Sample quantity	one piece	Sample state	/
Date of arrival	11/26/2018	Sample delivery personnel	Qiang Peng
Commission unit	Sichuan University	Manufacturing unit	Sichuan University
Commission unit address	No.29 Wangjiang Road, Chengdu, Sichuan, P. R. China.	Manufacturing unit address	No.29 Wangjiang Road, Chengdu, Sichuan, P. R. China.
Commission unit Zip code	610064	Manufacturing unit Zip code	610064
Commission unit Tel.	15828019886	Manufacturing unit Tel.	15828019886
Test Address	No 355, 2nd Tengfei Road, Southwest Airport Economic Development Zone, Chengdu, Sichuan, P. R. China.	Testing Period	11/27/2018
Test Standard	IEC60904-1:2006 Photovoltaic devices -Part 1: Measurement of Photovoltaic Current-Voltage Characteristics.		
Test conclusion	This column blank	(Special chapter for test report) Date of issue 11/28/2018	
Remarks	/		

Approved by [signature] Witnessed by [signature] Tested by [signature]

国家光伏产品质量监督检验中心 检验检测专用章

National Photovoltaic Product Quality Supervision & Inspection Center
TEST REPORT

Test Report No. AGXB118W00655

Test Results:

Clause	Test item(s)	Unit	Results
1	Current-voltage characteristics measurement	---	---
1.1	Open-circuit voltage, Voc	V	1.800
1.2	Short-circuit current, Isc	mA	0.451
1.3	Short-circuit current density, Jsc	mA/cm^2	11.287
1.4	Maximum-power, Pmax	mW	0.566
1.5	Maximum-power voltage, Vpmax	V	1.440
1.6	Maximum-power current, Ipmax	mA	0.393
1.7	Fill factor, FF	%	69.61
1.8	Conversion efficiency, η	%	14.14

Current Voltage characteristic at STC

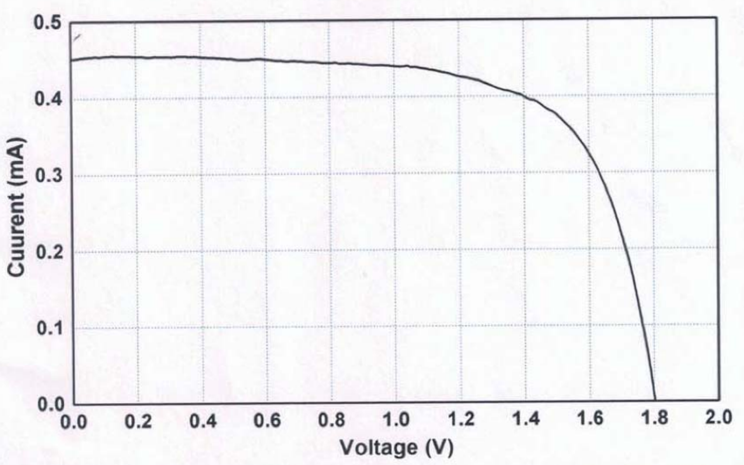

Remark: Sample was tested with a mask under the irradiation with a steady-state class calibrated AAA solar simulator. Working sample area (S) is determined by aperture outer range on the mask, S=4.00 mm^2.

----Blank----

Figure S14. Certification of the homo-tandem device based on PBDTSF-TZNT:IT-4F in National Photovoltaic Product Quality Supervision & Inspection Center of China. Related to Figure 6.

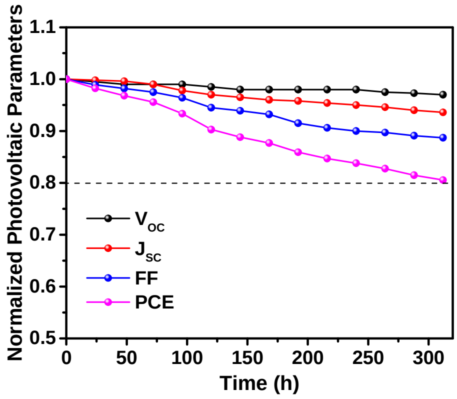

Figure S15. Normalized PCEs of the homo-tandem device based on PBDTSF-TZNT:IT-4F kept in glove box. Related to Figure 6.

Supplemental Tables

Table S1. Photovoltaic parameters of the PBDTSF-TZNT:IT-4F (1:1, w/w) devices processed from different solvents. Related to Figure 3 and Table 2.

solvent	Concentration (mg/mL)	V_{oc} (V)	J_{sc} (mA/cm^2)	J_{EQE} (mA cm^{-2})	FF (%)	PCE (%)
CF	14	0.96	17.02	16.79	64.1	10.48
CB	20	0.95	15.68	15.51	56.3	8.39
DCB	25	0.95	15.10	14.78	54.4	7.80

Table S2. Photovoltaic parameters of the PBDTSF-TZNT:IT-4F processed from CF with different D/A ratios and additive content. Related to Figure 3 and Table 2.

D/A	DIO (v%)	V_{oc} (V)	J_{sc} (mA/cm^2)	J_{EQE} (mA cm^{-2})	FF (%)	PCE (%)
1:0.5	-	0.94	15.83	15.44	57.1	8.50
1:1	-	0.96	17.02	16.79	64.1	10.48
1:1.5	-	0.95	16.57	16.16	62.2	9.79
1:1	0.1	0.94	17.78	17.25	65.6	10.97
1:1	0.2	0.94	18.41	18.10	70.1	12.13
1:1	0.3	0.93	18.25	17.83	66.8	11.34

Table S3. Photovoltaic parameters of the NF-PSCs treated with different thermal annealing temperatures. Related to Figure 3 and Table 2.

Active layer	Temp. (^{o}C)	V_{oc} (V)	J_{sc} (mA/cm^2)	J_{EQE} (mA cm^{-2})	FF (%)	PCE (%)
	r.t.	0.96	15.01	14.63	58.3	8.40
PBDTS-TZNT:ITIC	80	0.94	16.76	16.29	64.2	10.12
(1:1, CF, 0.2v%DIO)	100	0.94	16.92	16.45	65.7	10.45
	120	0.94	16.87	16.36	65.1	10.33
PBDTS-TZNT:IT-4F	r.t.	0.89	17.78	17.22	61.8	9.78
(1:1, CF, 0.2v%DIO)	80	0.88	18.43	18.01	67.1	10.89

	100	0.88	18.65	18.23	68.9	11.31
	120	0.88	18.58	18.12	67.9	11.10
	r.t.	1.00	16.76	16.31	65.5	10.98
PBDTSF-TZNT:ITIC	80	0.98	17.24	16.54	69.1	11.67
(1:1, CF, 0.2v%DIO)	100	0.98	17.58	17.22	70.6	12.16
	120	0.98	17.32	16.91	70.3	11.93
	r.t.	0.94	18.41	18.10	70.1	12.13
PBDTSF-TZNT:IT-4F	80	0.93	18.78	18.49	72.6	12.68
(1:1, CF, 0.2v%DIO)	100	0.93	19.23	18.97	74.1	13.25
	120	0.93	19.07	18.81	73.2	12.99

Table S4. Summarized parameters for the ordered molecular structures. Related to Figure 4.

Blend film	lamellar stacking	π-π stacking		
	d_l [Å] (q_{xy} [Å^{-1}])	d_π [Å] (q_z [Å^{-1}])	FWHM [Å^{-1}][a]	L_C [Å][b]
PBDTS-TZNT	25.1 (0.25)	3.61 (1.73)	0.27	23
PBDTSF-TZNT	25.1 (0.25)	3.59 (1.74)	0.25	25
PBDTS-TZNT:ITIC	25.1 (0.25)	3.65 (1.72)	0.39	16
PBDTS-TZNT:IT-4F	25.1 (0.25)	3.63 (1.73)	0.24	26
PBDTSF-TZNT:ITIC	25.1 (0.25)	3.63 (1.73)	0.28	22
PBDTSF-TZNT:IT-4F	25.1 (0.25)	3.61 (1.74)	0.21	30

[a]Full width at half-maximum (FWHM) for the (010) peak along the q_z axis.

[b]Coherent length estimated from the Scherrer's equation ($L_C = 2\pi/\text{FWHM}$) for the π-π stacking of the face-on crystallite.

Supplemental References

Beek, W.J., Wienk, M.M., Kemerink, M., Yang, X., and Janssen, R.A. (2005). Hybrid zinc oxide conjugated polymer bulk heterojunction solar cells. J. Phys. Chem. B *109*, 9505-9516.

Dong, Y., Hu, X.W., Duan, C.H., Liu, P., Liu, S.J., Lan, L.Y., Chen, D.C., Ying, L., Su, S.J., Gong, X., Huang, F., and Cao, Y. (2013). A series of new medium-bandgap conjugated polymers based on naphtho[1,2-c:5,6-c]bis(2-octyl-[1,2,3]triazole) for high-performance polymer solar cells. Adv. Mater. *25*, 3683-3688.

Zhang, G.J., Xu, X.P., Bi, Z.Z., Ma, W., Tang, D.S., Li, Y., and Peng, Q. (2018). Fluorinated and alkylthiolated polymeric donors enable both efficient fullerene and nonfullerene polymer solar cells. Adv. Funct. Mater. *28*, 1706404.
